# Ginkgolides protect against amyloid-β_1–42_-mediated synapse damage *in vitro*

**DOI:** 10.1186/1750-1326-3-1

**Published:** 2008-01-07

**Authors:** Clive Bate, Mourad Tayebi, Alun Williams

**Affiliations:** 1 Department of Pathology and Infectious Diseases, Royal Veterinary College, Hawkshead Lane, North Mymms, Herts, AL9 7TA, UK

## Abstract

**Background:**

The early stages of Alzheimer's disease (AD) are closely associated with the production of the Aβ_1–42 _peptide, loss of synapses and gradual cognitive decline. Since some epidemiological studies showed that EGb 761, an extract from the leaves of the *Ginkgo biloba *tree, had a beneficial effect on mild forms of AD, the effects of some of the major components of the EGb 761 extract (ginkgolides A and B, myricetin and quercetin) on synapse damage in response to Aβ_1–42 _were examined.

**Results:**

The addition of Aβ_1–42 _to cortical or hippocampal neurons reduced the amounts of cell associated synaptophysin, a pre-synaptic membrane protein that is essential for neurotransmission, indicating synapse damage. The effects of Aβ_1–42 _on synapses were apparent at concentrations approximately 100 fold less than that required to kill neurons; the synaptophysin content of neuronal cultures was reduced by 50% by 50 nM Aβ_1–42_. Pre-treatment of cortical or hippocampal neuronal cultures with ginkgolides A or B, but not with myrecitin or quercetin, protected against Aβ_1–42_-induced loss of synaptophysin. This protective effect was achieved with nanomolar concentrations of ginkgolides. Previous studies indicated that the ginkgolides are platelet-activating factor (PAF) receptor antagonists and here we show that Aβ_1–42_-induced loss of synaptophysin from neuronal cultures was also reduced by pre-treatment with other PAF antagonists (Hexa-PAF and CV6209). PAF, but not lyso-PAF, mimicked the effects Aβ_1–42 _and caused a dose-dependent reduction in the synaptophysin content of neurons. This effect of PAF was greatly reduced by pre-treatment with ginkgolide B. In contrast, ginkgolide B did not affect the loss of synaptophysin in neurons incubated with prostaglandin E_2_.

**Conclusion:**

Pre-treatment with ginkgolides A or B protects neurons against Aβ_1–42_-induced synapse damage. These ginkgolides also reduced the effects of PAF, but not those of prostaglandin E_2_, on the synaptophysin content of neuronal cultures, results consistent with prior reports that ginkgolides act as PAF receptor antagonists. Such observations suggest that the ginkgolides are active components of *Ginkgo biloba *preparations and may protect against the synapse damage and the cognitive loss seen during the early stages of AD.

## Background

Alzheimer's disease (AD) is a complex and genetically heterogeneous disease that is the most common form of dementia and affects up to 15 million individuals worldwide. The amyloid hypothesis of AD pathogenesis maintains that the primary event is the production and accumulation of amyloid-β (Aβ) peptides, derived from abnormal proetolytic cleavage of the amyloid precursor protein [1-3]. The accumulation of Aβ peptides leads to the subsequent disruption of neuronal processes, abnormal phosphorylation of tau and ultimately the dysfunction and death of neurons. However, the precise mechanisms by which Aβ peptides lead to neuronal damage remain to be fully determined. Initially it was thought that fibril formation by Aβ peptides was required for neurotoxicity [4], however, more recent studies showed that smaller soluble oligomers of Aβ or Aβ-derived diffusible ligands are also potent neurotoxins [5,6].

The early stages of AD are characterised by memory impairment and subtle behavioural changes, associated with changes in synaptic function and a reduction in the levels of synaptophysin, a presynaptic membrane protein essential for neurotransmitter release and the recycling of synaptic vesicles [7], within the brain. These occur before any gross neurological damage is observed [8-10]. The loss of synapses and the reduction in synaptophysin levels are features of AD that strongly correlate with cognitive decline [11]. We previously developed an *in vitro *model to examine the effects of Aβ peptides on synapses where the amounts of synaptophysin in neuronal cultures were measured as a surrogate marker of synapse function. The addition of Aβ_1–42 _reduced the synaptophysin content of neurons indicating the loss of synapses in these cultures [12]. In this paper, a possible mechanism leading to Aβ_1–42_-induced loss of synaptophysin from neuronal cultures was investigated.

Extracts from the leaves of the *Ginkgo biloba *tree are becoming increasingly popular as a treatment that is claimed to reduce memory loss and the symptoms of mild cognitive disorders including AD [13-15]. However, there remains considerable controversy regarding the mechanisms of action of these preparations, or even whether such preparations have any clinical benefit. While some published studies conclude that the use of a standardized extract of the leaves of the *Ginkgo biloba *tree (EGb 761) reduces the symptoms of mild cognitive disorders including AD [13,16], other studies have failed to show clinical benefit [17]. Since the EGb 761 extract contains many compounds, including ginkgolides and the flavonoglycosides myricetin and quercetin, it is not clear which individual components of this extract are efficacious. Our previous studies showed that pre-treatment with ginkgolides protected against Aβ_1–42_-induced neuronal death [18] and in the current study we tested the main compounds in the EGb 761 extract on cultured neurons and for their effects on synapse damage in response to Aβ_1–42_. We report that pre-treatment of cultured neurons with ginkgolides A or B significantly reduced the effects of Aβ_1–42 _on synapses.

## Results

### Ginkgolides protect against Aβ_1–42_-induced synapse damage

**Figure 1:**
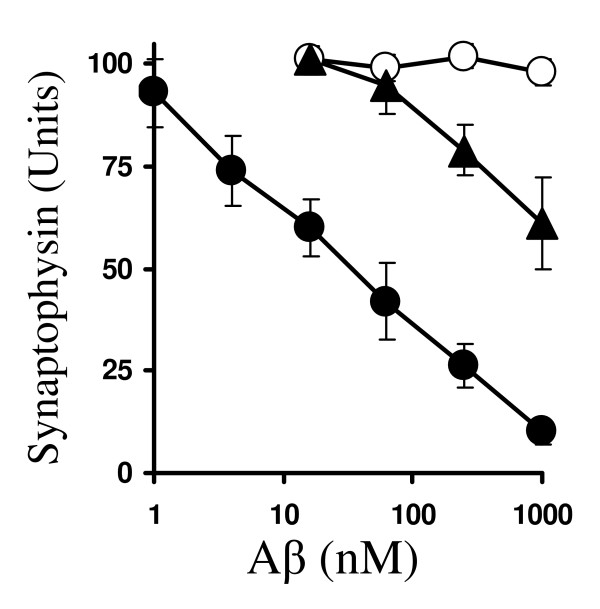
**Aβ_1–42 _causes a dose-dependent reduction in the synaptophysin content of neurons**. The synaptophysin content of cortical neuronal cultures treated for 24 hours with varying concentrations of Aβ_1–42 _(solid circle), Aβ_42-1 _(hollow circle) or Aβ_1–40 _(solid triangle). Values shown are the mean synaptophysin content ± SD from 12 observations.

Here we report that both Aβ_1–42 _and Aβ_1–40 _peptides, but not the control peptide Aβ_42-1_, reduced the synaptophysin content of cortical neurons in a dose-dependent manner. While the synaptophysin content of cortical neurons was reduced by 50% by 50 nM Aβ_1–42_, much higher amounts of Aβ_1–40 _(2 μM) were required to have the same effect (Figure 1). The effects of Aβ peptides on synapses were apparent at concentrations that did not affect neuronal survival. Since Aβ_1–42 _was the more potent peptide at damaging synapses, all further experiments were carried out using this peptide. Greater than 90% of this activity remained after Aβ_1–42 _preparations were passed through a 100 Kd filter, or after high speed centrifugation (data not shown), suggesting that the Aβ species responsible were small soluble oligomers or proto-fibrils rather than the larger insoluble fibrils which this peptide sometimes forms.

**Figure 2:**
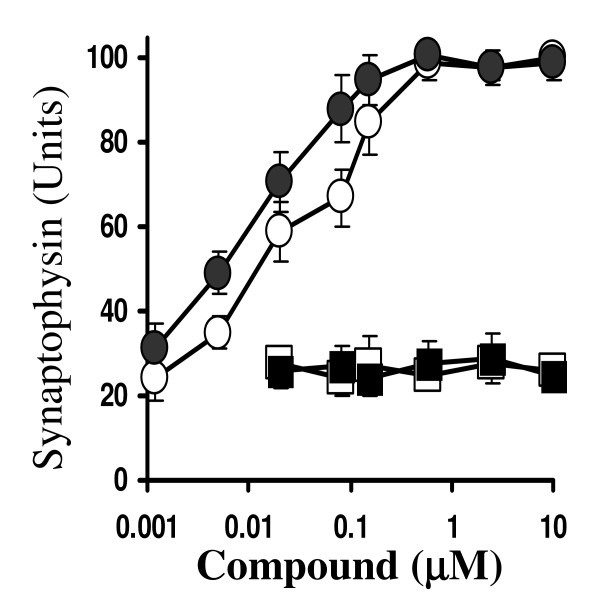
**Effects of individual *Ginkgo biloba *components on Aβ_1–42_-induced loss of synaptophysin**. The synaptophysin content of cortical neuronal cultures pre-treated for 3 hours with varying concentrations of ginkgolides A (hollow circle) or B (solid circle), myricetin (solid squares) or quercetin (hollow squares) and subsequently incubated with 200 nM Aβ_1–42 _for 24 hours. Values shown are the mean synaptophysin content ± SD from 6 observations.

To determine if treatment with individual components of *Gingko biloba *extracts could modify the effects of Aβ_1–42_, cortical neurons were pre-treated with different concentrations (0.001 to 10 μM) of the ginkgolides A or B, or the flavanoglycosides (quercetin or myricetin) before the addition of 200 nM Aβ_1–42_. Treatment of neuronal cultures with these concentrations of ginkgolides A or B, myricetin or quercetin did not affect their synaptophysin content showing that these compounds alone do not damage synapses or stimulate synaptogenesis. Pre-treatment with either ginkgolide A or B reduced the effects of Aβ_1–42 _on the synaptophysin content of neuronal cultures (Figure 2). These effects were dose-dependent and the ED_50 _(the concentration of drug that increased synaptophysin content by 50% in Aβ_1–42_-treated neurons) was 40 nM for ginkgolide A and 10 nM for ginkgolide B. Pre-treatment with either quercetin or myricetin did not affect the loss of synaptophysin induced by Aβ_1–42_.

**Figure 3:**
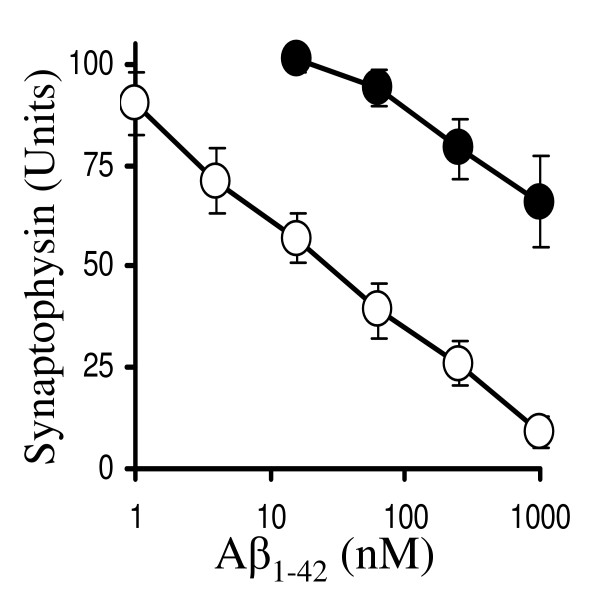
**Ginkgolide B protects cortical neurons against Aβ_1–42_-induced loss of synaptophysin**. Primary cortical neurons were pre-treated for 3 hours with control medium (solid circle) or with 1 μM ginkgolide B (hollow circle) and subsequently incubated for 24 hours with different concentrations of Aβ_1–42 _as shown. Each point represents the mean synaptophysin content ± SD from 9 observations.

The synaptoprotective effect of ginkgolide B was competitive; while the concentration of Aβ_1–42 _required to reduce the synaptophysin content of untreated neurons by 50% was 50 nM, 8 μM Aβ_1–42 _was required to have the same effect in neurons treated with 1 μM ginkgolide B (Figure 3). This effect of ginkgolide B required pre-treatment; there was no significant difference between the amount of synaptophysin in neuronal cultures incubated with 200 nM Aβ_1–42 _and neuronal cultures in which 1 μM ginkgolide B was added to neurons 30 minutes after the addition of 200 nM Aβ_1–42 _(27 units ± 5 v 30 ± 4, n = 6, P > 0.05).

**Figure 4:**
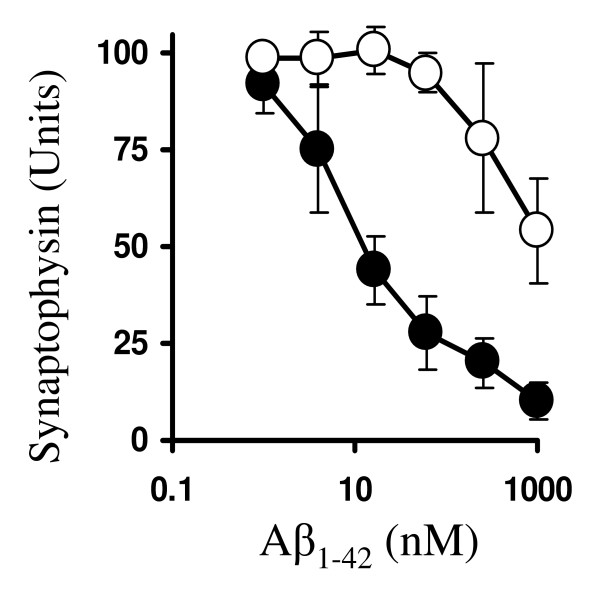
**Ginkgolide B protects hippocampal neurons against Aβ_1–42_-induced loss of synaptophysin**. The synaptophysin content of untreated hippocampal neurons (solid circle) or hippocampal neurons pre-treated for 3 hours with 1 μM ginkgolide B (hollow circle) and subsequently incubated with different concentrations of Aβ_1–42 _for 24 hours as shown. Values shown are the mean synaptophysin content ± SD from 9 observations.

The effects of ginkgolide B were not confined to cortical neurons; the amounts of synaptophysin in hippocampal neuronal cultures were also reduced by Aβ_1–42_. The amount of Aβ_1–42 _required to reduce the synaptophysin content of hippocampal neuronal cultures by 50% was 10 nM. Pre-treatment with 1 μM ginkgolide B resulted in Aβ_1–42 _having a significantly reduced effect on the amounts of synaptophysin in hippocampal cultures; in these cultures the amount of Aβ_1–42 _required to reduce synaptophysin content by 50% was increased to 4 μM (Figure 4).

### Ginkgolides do not affect the binding of Aβ_1–42 _to neurons

**Figure 5:**
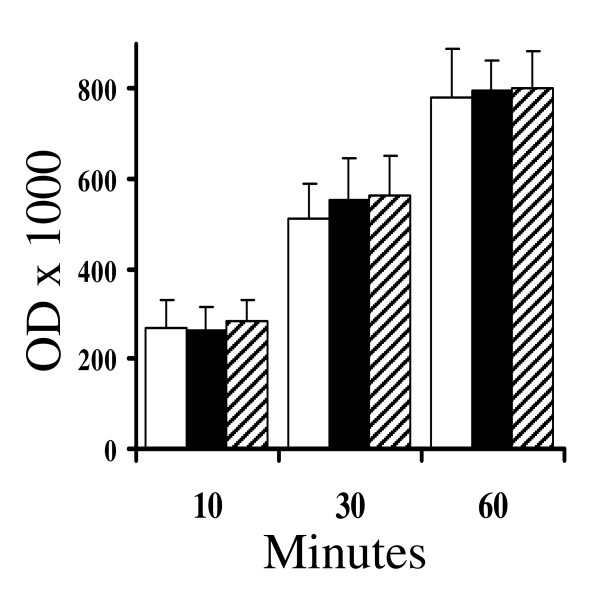
**Ginkgolides do not affect the incorporation of Aβ_1–42_ into neurons**. The amounts of biotinylated-Aβ_1–42 _in cell extracts from untreated neurons (hollow bars) or neurons pre-treated for 3 hours with ginkgolide A (solid bars) or ginkgolide B (striped bars) and subsequently incubated with 200 nM biotinylated-Aβ_1–42 _for the time periods shown. Each point represents the mean amount of bound biotinylated-Aβ_1–42 _(optical density × 1000) ± SD from 9 observations.

The hypothesis that ginkgolide-treated neurons bind less Aβ_1–42 _was tested by treating cortical neurons with biotinylated-Aβ_1–42_. Firstly, we tested whether biotinylation affected the activity of the Aβ_1–42 _peptide; there were no significant differences in the synaptophysin content of neuronal cultures treated with 200 nM Aβ_1–42 _and 200 nM biotinylated-Aβ_1–42 _(28 units ± 4 v 27 ± 5, n = 6, P > 0.05). Next, cortical neurons were treated with 1 μM ginkgolides A or B prior to the addition of 200 nM biotinylated-Aβ_1–42_. Neuronal extracts were collected at different time points after the addition of peptide and the amounts of cell bound biotinylated-Aβ_1–42 _were measured. The amounts of biotinylated-Aβ_1–42 _increased in a time-dependent manner over the first hour, but showed no significant increases thereafter. There were no significant differences in the amounts of biotinylated-Aβ_1–42 _ingested by untreated cortical neurons and neurons treated with 1 μM ginkgolides A or B (Figure 5).

### PAF mimics the effects of Aβ_1–42 _on synaptophysin levels

**Figure 6:**
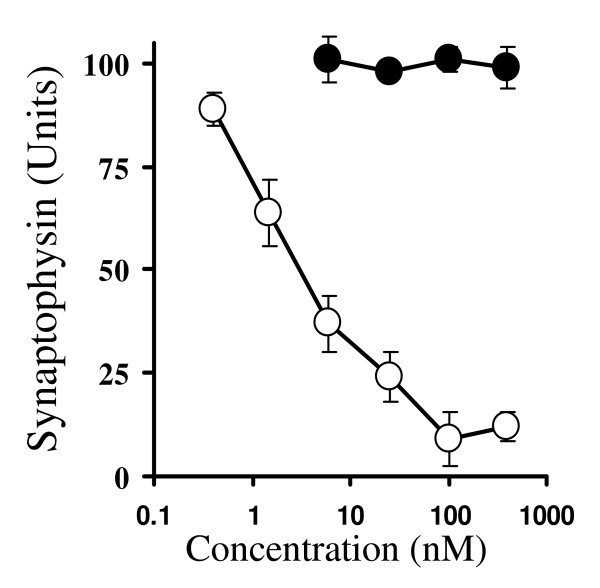
**PAF, but not lyso-PAF, causes a dose-dependent loss of synaptophysin from neurons**. The amounts of synaptophysin in cortical neurons treated with different concentrations of PAF (hollow circle) or lyso-PAF (solid circle) for 24 hours. Each point represents the mean synaptophysin content ± SD from 9 observations.

**Figure 7:**
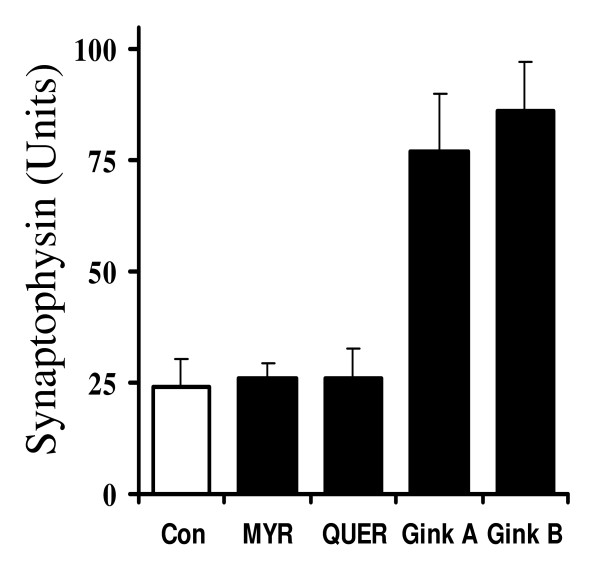
**Ginkgolides A and B protect against PAF-mediated synapse damage**. The synaptophysin content of untreated neurons (Con) (hollow squares) or neuronal cultures pre-treated for 3 hours with 1 μM ginkgolides A or B or 25 μM myricetin or quercetin (solid squares) as shown and subsequently incubated with 25 nM PAF for 24 hours. Values shown are the mean synaptophysin content ± SD from 6 observations.

Ginkgolides have been reported to antagonise PAF receptors [19] raising the possibility that PAF mediates the synapse damage caused by Aβ_1–42_. The addition of PAF, but not lyso-PAF, mimicked the effects of Aβ_1–42_, and caused a dose-dependent reduction in the synaptophysin content of cortical neurons (Figure 6). The synaptophysin content of cortical neurons was reduced by 50% by 5 nM PAF, a concentration that did not affect neuronal survival. Pre-treatment of neurons with 1 μM ginkgolides A or B, but not with 25 μM myricetin or 25 μM quercetin, reduced the effects of PAF on the synaptophysin content of neuronal cultures (Figure 7).

**Figure 8:**
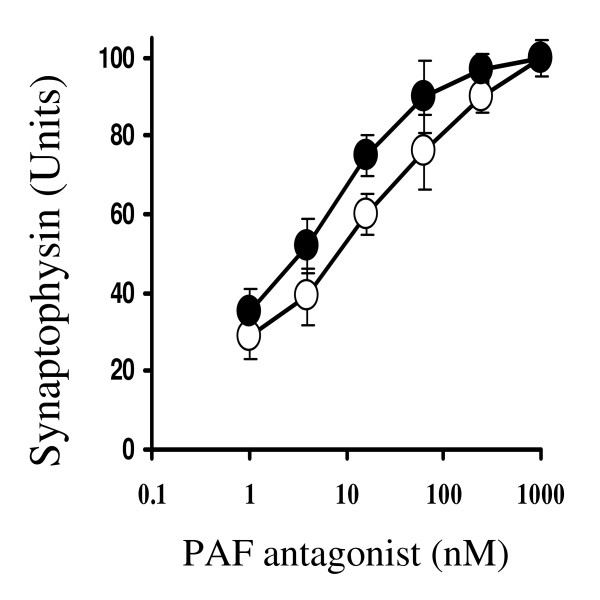
**PAF antagonists protect against Aβ_1–42 _induced synapse damage**. Cortical neurons were pre-treated with different concentrations of the PAF antagonists Hexa-PAF (solid circle) or CV6209 (hollow circle) for 3 hours prior to the addition of 100 nM Aβ_1–42 _for 24 hours. Values shown are the mean synaptophysin content, ± SD, of 9 observations.

These results are consistent with the hypothesis that ginkgolides protect synapses via antagonism of PAF receptors. Confirmation of this hypothesis was sought; pre-treatment with the PAF antagonists, hexa-PAF or CV6209 also caused a dose-dependent increase in the amounts of synaptophysin in neuronal cultures treated with 200 nM Aβ_1–42 _(Figure 8). Next we examined the effects of pre-treatment with a combination of ginkgolide B and hexa-PAF. The amounts of synaptophysin in neuronal cultures incubated with 200 nM Aβ_1–42 _was significantly increased by pre-treatment with either 10 nM ginkgolide B (25 units ± 7 v 58 ± 3, n = 6, P < 0.05) or with 10 nM hexa-PAF B (25 units ± 7 v 67 ± 6, n = 6, P < 0.05). Pre-treatment with the combination of 10 nM ginkgolide B and 10 nM hexa-PAF increased the synaptophysin content of cultures (74 units ± 4).

### Ginkgolide B does not protect against PGE_2_-induced synapse damage

Since PAF has been shown to increase the production of PGE_2 _from neurons *in vitro *[20] the effects of PGE_2 _on synapses was examined. The synaptophysin content of cortical neurons was significantly reduced following the addition of PGE_2_, or with two prostanoid E (EP) receptor agonists (butaprost or misoprostol). Pre-treatment of neurons with 1 μM ginkgolide B did not protect against the loss of synaptophysin in neuronal cultures incubated with 40 nM PGE_2_, 1 μM butaprost or 1 μM misoprostol. In contrast, the effects of PAF or Aβ_1–42 _on the synaptophysin content of neuronal cultures were significantly reduced by pre-treatment with 100 nM AH13205, an EP receptor antagonist (Table 1).

**Table 1:** Ginkgolide B does not affect PGE_2_-induced loss of synaptophysin.

	**Synaptophysin content (units)**		
	**Pre-treatment**		

	**None**	**Ginkgolide B (1 μM)**	**AH13205 (100 nM)**
**None**	100 ± 5	101 ± 3	102 ± 3
**Aβ_1–42 _(200 nM)**	24 ± 4	93 ± 7	97 ± 6
**PAF (10 nM)**	33 ± 7	78 ± 5	89 ± 8
**PGE_2 _(40 nM)**	41 ± 6	44 ± 7	96 ± 4
**Butaprost (1 μM)**	29 ± 5	37 ± 11	94 ± 7
**Misprostal (1 μM)**	32 ± 7	34 ± 6	98 ± 5

The effects of pre-treatment with ginkgolide B, or with the EP receptor antagonist AH13205 on the synaptophysin content of neuronal cultures incubated for 24 hours with Aβ_1–42_, PAF, PGE_2_, or with the EP receptors agonists butaprost or misoprostol as shown. Values shown are the mean synaptophysin content ± SD from 9 observations.

## Discussion

An in *vitro *model of Aβ-mediated synapse damage was used to investigate the effects of individual components of the *Gingko biloba *extract EGb 761 on the synapse damage that occurs in AD. The addition of Aβ_1–42_, and to a lesser extent Aβ_1–40_, reduced the synaptophysin content of cortical and hippocampal neuronal cultures. The hippocampus is thought to be involved in the formation of short term memory, the loss of which is one of the earliest clinical symptoms of AD [10]. In all assays, the effects of Aβ peptides on the synaptophysin content of neuronal cultures occurred at concentrations significantly lower than those required to kill neurons. These observation are consistent with *in vivo *observations that show that a loss of synapses and a reduction in synaptophysin levels occurs before any gross neurological damage is observed [8-11].

Although previous studies suggest that the flavonoglycosides have protective properties against oxidative stress *in vitro *[14,21], no protective effects of myricetin or quercetin against Aβ_1–42_-induced synapse damage were observed in these studies. Moreover, flavonoglycosides have limited bioavailability after oral administration [14] suggesting that these compounds are less likely to be responsible for the protective effects of the EGb 761 extract. In contrast, nanomolar concentrations of both ginkgolides A and B reduced the loss of synaptophysin induced by Aβ_1–42 _in cortical neuronal cultures. Pre-treatment with 1 μM ginkgolide B increased the amounts of Aβ_1–42 _required to reduce the synaptophysin content of cortical neurons by 50% from 50 nM in untreated neurons to 8 μM in ginkgolide B-treated neurons. The addition of Aβ_1–42 _also damaged synapses in cultured hippocampal neurons; it required only 10 nM Aβ_1–42 _to reduce the synaptophysin content of these cultures by 50%. While it is tempting to suggest that hippocampal neuronal synapses are more sensitive to Aβ_1–42 _than cortical neuronal synapses a direct comparison is invalid considering the differences in the ages of mice used and the number of contaminating astroglial cells. In hippocampal neuronal cultures pre-treated with ginkgolide B over 1 μM Aβ_1–42 _was needed to reduce the synaptophysin content by 50%.

The mechanism of the protective effect of ginkgolides was sought. We dismissed the simplest explanation, that ginkgolides reduce the ability of neurons to bind Aβ peptides, because ginkgolide-treated neurons ingested similar amounts of biotinylated-Aβ_1–42 _as untreated neurons. Aβ_1–42 _peptides exist in different states ranging from small soluble oligomers or Aβ-derived diffusible ligands [5,6], to larger fibrillar structures [4]. Therefore it is possible that ginkgolides bind directly to Aβ_1–42 _peptides and promote the formation of an inactive conformer. Although this hypothesis cannot be totally excluded, ginkgolide-treated cultures that had been washed (which removed any non-bound ginkgolides) remained resistant to the synapse damage induced by Aβ_1–42_. We note with interest an observation that Aβ fibrillogenesis is accelerated in the presence of plasma, endosomal and lysosomal membranes, but reduced in Golgi membranes. The authors suggested that the composition of these membranes affected Aβ oligomerisation [22] and the possibility that ginkgolides affect membrane composition and subsequently Aβ fibril formation within intracellular compartments cannot be excluded.

Since Aβ peptides activate phospholipase A_2 _(PLA_2_) [23], a major step in the production of PAF [24], the possibility that synapse damage occurs as a consequence of Aβ_1–42_-induced production of PAF was investigated. PAF receptors are present in synapses [25] and PAF is required for synapse maintenance [26] and long term potential in the hippocampus [27]. However, higher concentrations of PAF are implicated in neurodegenerative diseases including AD [28]. Here we report that the addition of PAF caused a dose-dependent reduction in the synaptophysin content of cortical neurons; 5 nM PAF reduced synaptophysin content by 50%. The effects of PAF are transmitted via a G-protein coupled receptor [29] and the addition of lyso-PAF, a non-acetylated structural analogue of PAF that does not bind to the PAF receptor, did not cause synapse damage. One of the many reported effects of the ginkgolides is antagonism of PAF receptors [19] and in the current study we demonstrated that ginkgolide-treated neurons were partially resistant to the effects of PAF, as well as that of Aβ_1–42_, on synapses. The synapse-protective effect of ginkgolide B was consistently stronger than ginkgolide A, consistent with reports that ginkgolide B has greater affinity for PAF receptors than ginkgolide A [30]. Pre-treatment with two non-related PAF antagonists, hexa-PAF and CV6209 [31,32], also protected synapses suggesting that antagonism of the PAF receptors mediates the synaptoprotective effects of the ginkgolides. Although pre-treatment with a combination of ginkgolide B and hexa-PAF increased the synaptophysin content of cultures relative to treatment with ginkgolide B or hexa-PAF alone, their affects appeared to be additive rather than synergistic.

Both Aβ_1–42 _and PAF, but not lyso-PAF, increased the production of PGE_2 _in cortical neurons *in vitro *[20] raising the possibility that the effects of PAF on synapses were mediated through the production of PGE_2_. While pre-treatment with ginkgolide B reduced both Aβ_1–42 _and PAF-induced production of PGE_2_, it did not alter the effects of PGE_2_, or two EP receptor agonists (butaprost or misoprostol), on the synaptophysin content of neuronal cultures. In contrast, the EP receptor antagonist AH13205 [33] protected against Aβ_1–42_, PAF and PGE_2_-induced synaptophysin loss. These results are consistent with the hypothesis that ginkgolides protect against Aβ_1–42_-mediated synapse damage by modifying the production of PGE_2_. This hypothesis is also consistent with epidemiological data that show that the use of cyclo-oxygenase inhibitors reduce the severity of AD [34]. We suggest that activation of PLA_2 _by Aβ_1–42 _leads to sustained production of PAF which in turn increases PGE_2 _production, and that high concentrations of PGE_2 _damaged synapses. PGE_2 _modulates synaptic transmission [35] and although high concentrations were reported to induce apoptosis in hippocampal neurons [36], we found that 10 μM PGE_2 _did not kill cortical neurons (data not shown). Similarly we found that although the EP receptor antagonist AH13205 reduced Aβ_1–42_-induced loss of synaptophysin it did not affect Aβ_1–42_-induced neuronal death (data not shown).

It is worth noting that for many *Ginkgo biloba *extracts, extraction procedures are used that optimise the flavonoglycoside content. However, these procedures may result in extracts that contain different amounts of ginkgolides, and a recent analysis of commercial *Ginkgo biloba *supplements by liquid chromatography/mass spectrometry demonstrated remarkable variations in ginkgolide content [37]. Therefore, variations in the ginkgolide content of different extracts may be a factor that explains the variability of results obtained in clinical studies.

## Conclusion

In summary we show that sub-toxic concentrations of Aβ_1–42 _damaged synapses in cultured cortical and hippocampal neurons. We propose that these changes model the early stages of AD, where there are significant behavioural changes including synaptic dysfunction and alterations in the development of short-term memory, but before gross neuronal loss is observed [38]. We report that neurons treated with ginkgolides A or B show increased resistance to the effects Aβ_1–42 _on synapses. This effect of the ginkgolides correlated with their ability to antagonise PAF receptors [30], and in these studies PAF also reduced the synaptophysin content of cortical neuronal cultures. Recent studies showed that the bioavailability of ginkgolides after oral administration is high [14] suggesting that the ginkgolides have the potential to reduce synapse damage in AD should they be able to cross the blood brain barrier and penetrate the central nervous system.

## Methods

### Neuronal cultures

Cortical neurons were prepared from the brains of day 15.5 C57BL/6J mouse embryos. After mechanical dissociation, cell sieving and isolation on histopaque (Sigma, Poole, UK), neuronal precursors were plated at 2 × 10^5 ^cells/well in 48 well plates in Hams F12 containing 5% foetal calf serum (FCS) for 2 hours. Cultures were then shaken (600 r.p.m for 5 minutes) and non-adherent cells removed by 2 washes in phosphate buffered saline (PBS). Neurons were grown in neurobasal medium (NBM) containing B27 components (Invitrogen, Paisley, UK) for 7 days. Immunostaining showed that the cells were greater than 97% neurofilament positive. Less than 3% stained for GFAP (astrocytes) or for F4/80 or CD14 (microglial cells).

Hippocampal neuronal cultures were prepared from the brains of adult C57BL/6J mice as previously described [39]. Briefly, hippocampi were dissected from the adult brain tissue and triturated with fire-polished pasteur pipette in Hams F12 containing 5% FCS, 0.35% glucose, 0.025% trypsin, and 0.1% type IV collagenase (Invitrogen). After 30-minute incubation at 37°C, the cells were shaken and the cell suspension was passed through a 100 μM cell strainer (Becton Dickenson). Cells were collected by centrifugation, washed twice in Hams F12 containing 5% FCS, and plated at 2 × 10^5 ^cells/well in 48 well plates for 24 hours. Cultures were shaken (600 r.p.m for 5 minutes) to remove non-adherent cells, washed twice with PBS and the remaining cells were cultured in NBM containing B27 components and 10 ng/ml glial-derived neurotrophic factor (Sigma) for 7 days. Immunostaining showed that after 7 days cultured in NBM less than 5% of the viable cells stained for GFAP or F4/80 (astrocytes or microglial cells). Both types of neuronal cultures were pre-treated with test compounds for 3 hours. Cells were then washed with PBS 3 times to remove unbound test compounds before the addition of Aβ peptides in NBM/B27 components; the amounts of synaptophysin in treated cultures were measured 24 hours later.

### Cell extracts

Treated neurons were washed 3 times with PBS and homogenised in a buffer containing 150 mM NaCl, 10 mM Tris-HCl, 10 mM EDTA, 0.2% SDS and mixed protease inhibitors (AEBSF, Aprotinin, Leupeptin, Bestain, Pepstatin A and E-46) (Sigma) at 1 × 10^6 ^cells/ml. Nuclei and cell debris was removed by centrifugation (300 × *g *for 5 minutes).

### Synpatophysin ELISA

Synaptophysin levels in neuronal extracts were measured by a sandwich ELISA as previously described [12]. In short, the capture antibody (0.5 μg/ml) was a mouse monoclonal antibody (mab) anti-synaptophysin MAB368 (Chemicon, UK) and bound synaptophysin was detected using a goat polyclonal anti-synaptophysin (Abcam, Chandler's Ford, UK). Bound antibodies were detected with a biotinylated anti-goat IgG (Dako, Cambridgeshire, UK), extravidin-alkaline phosphatase and 1 mg/ml 4-nitrophenol phosphate in diethanolamine buffer (Sigma). Absorbance was measured on a microplate reader at 450 nM and the synaptophysin content was calculated by reference to a standard curve. Samples were expressed as "units synaptophysin" where 100 units was arbitrarily defined as the amount of synaptophysin in 1 × 10^6 ^untreated cells. A standard curve was generated from this sample using sequential log 2 dilutions (range 100 to 1.56 units).

### Ingestion of biotinylated-Aβ_1–42_

Cortical neurons were pre-treated with drugs for 3 hours and washed 3 times with PBS prior to the addition of biotinylated-Aβ_1–42_. After 10, 30 or 60 minutes neurons were washed a further three times with warm PBS to remove unbound peptide and then homogenised in a buffer containing 150 mM NaCl, 10 mM Tris-HCl, 10 mM EDTA, 0.2% SDS and mixed protease inhibitors at 1 × 10^6 ^cells/ml. Nuclei and cell debris was removed by low speed centrifugation (300 × *g *for 5 minutes). The amounts of biotinylated-Aβ_1–42 _in cell extracts were determined by ELISA. All stages of the ELISA were carried out at 37°C for 1 hr. Maxisorb Immunoplates (Nunc, Roskilde, Denmark) were coated with 0.1 μg mouse mab to Aβ_1–42 _(Immunodiagnostic Systems, Tyne and Wear, UK) as a capture antibody. Samples were applied and the amounts of biotinylated-Aβ_1–42 _bound were determined by incubation with extravidin-alkaline phosphatase and 1 mg/ml 4-nitrophenol phosphate in diethanolamine buffer. Absorbance was measured at 450 nM and the amounts of biotinylated-Aβ_1–42 _in samples were expressed as optical density (OD) × 1000.

### Peptides

A peptide corresponding to amino acids 1 to 42 of the Aβ protein (Aβ_1–42_), a biotinylated version containing biotin at the terminal aspartic acid residue (biotinylated-Aβ_1–42_), Aβ_1–40 _and a control peptide (Aβ_42-1_) were obtained from Bachem (St Helens, UK). 1 mM stock solutions of peptide were kept at -70°C, thawed on the day of use and sonicated prior to dilution and addition to cells.

### Drugs

CV6209, ginkgolide B, myricetin, quercetin, platelet-activating factor (PAF), lyso-PAF, butaprost, misoprostol and AH13025 were obtained from Sigma. Ginkgolide A, hexa-PAF and PGE_2_, were obtained from Calbiochem (Nottingham, UK).

### Statistical Analysis

Comparison of treatment effects were carried out using one and two way analysis of variance techniques as appropriate. *Post hoc *comparisons of means were performed as necessary.

## Abbreviations

Alzheimer's disease (AD), 

amyloid-β (Aβ), 

enzyme-linked immunoassay (ELISA), 

cyclo-oxygenase (COX), 

a standardized extract of the leaves of the *Ginkgo biloba *tree (EGb 761), 

fetal calf serum (FCS), 

monoclonal antibody (mab), 

phospholipase A_2 _(PLA_2_), 

platelet-activating factor (PAF), 

prostaglandin (PG).

## Competing interests

The author(s) declare that they have no competing interests.

## Authors' contributions

CB was responsible for the conception, planning of performance of experiments, and for writing this manuscript. Both MT and AW contributed to the planning of experiments, interpretation of results and the writing of the manuscript. All authors have read and approved the manuscript.
